# Feeding/Eating Problems in Children Who Refrained From Treatment in the Past: Who Did (Not) Recover?

**DOI:** 10.3389/fped.2022.860785

**Published:** 2022-05-03

**Authors:** Eric Dumont, Anita Jansen, Pieter C. Duker, Daniel M. Seys, Nick J. Broers, Sandra Mulkens

**Affiliations:** ^1^Department of Research and Development, SeysCentra, Malden, Netherlands; ^2^Department of Clinical Psychological Science, Faculty of Psychology and Neuroscience, Maastricht University, Maastricht, Netherlands; ^3^Department of Psychiatry & Neuropsychology, Faculty of Health, Medicine, and Life Sciences, Maastricht University, Maastricht, Netherlands; ^4^Department of Methodology & Statistics, Faculty of Psychology and Neuroscience, Maastricht University, Maastricht, Netherlands

**Keywords:** Avoidant/Restrictive Food Intake Disorder, children, predictors, feeding or eating problems, behavioral treatment

## Abstract

**Background:**

Young children with disordered feeding may be at increased risk for problematic eating in the future. This retrospective study attempts to identify predictors of later feeding problems.

**Objectives:**

Children (*N* = 236) with disordered feeding, who refrained from behavioral treatment after consultation at a tertiary treatment center for feeding and eating problems were followed-up after, on average, 6 years and 3 months (timepoint 2).

**Method:**

Logistic regressions were carried out with characteristics taken at intake (timepoint 1)—sex, pre/dysmaturity, gastro-intestinal disease, history of age-adequate feeding, syndrome/developmental impairment, autism spectrum disorder, comorbidity, age, and several variables of a restrictive- and selective food intake—and duration between timepoint 1 and 2, as predictor variables, and age-appropriate food intake at *t*2 as the dependent variable.

**Results:**

Despite improvement over time, 63% did *not* reach an age-adequate food intake at *t*2. Predictors of age-*in*adequate food intake were: (a) older age; (b) sex (male), (c) longer duration between timepoint 1 and timepoint 2; (d) autism spectrum disorder; (e) selective texture choices and (f) lack of varied nutritional intake.

**Conclusion:**

This study shows that most untreated young children's feeding problems do not improve over years. Besides the advice to seek help at an early age, it seems especially recommended to treat (male) children with autism spectrum disorder and selective feeding patterns.

## Introduction

Disordered feeding and eating can take various forms, such as refusal of any orally provided food, acceptance of only particular sorts of food and/or eating very small amounts of food. Disordered feeding and eating in childhood can develop into an extremely selective food intake and/or restriction, referred to as an “age-inappropriate feeding pattern,” or even a serious feeding or eating disorder such as Avoidant/Restrictive Food Intake Disorder (ARFID). Due to the wide range of these disordered feeding and eating patterns, as well as differences in severity and appearance, prevalence numbers vary. Cardona Cano and colleagues (1) found - among the Dutch population - prevalence numbers from 26.5% at an age of 1.5 years, up to 27.6% at age 3, with a decline to 13.2% at age 6, in picky/fussy eating behavior. Prevalence of more extremely disordered feeding or eating patterns in 8 to 18-year-olds can range from 1.5 to 3.2% (2, 3).

The previous edition of the Diagnostic and Statistical Manual of Mental Disorders (4) classified the above-mentioned problems as “Feeding Disorder of Infancy or Early Childhood,” while the latest edition, DSM-5 (5) adjusted the classification to Avoidant/Restrictive Food Intake Disorder (ARFID), and included this in the chapter of feeding and eating disorders. Compared to DSM-IV, major changes concern specific representations of ARFID, more specific parameters, as well as an extension of the age limit (6–9). That is, ARFID can manifest in infancy or (early) childhood but can also develop in late adulthood, whereas the DSM-IV-TR required the diagnosis to be made before the age of six. ARFID is associated with one or more of the following: substantial weight loss or insufficient growth, nutritional deficiency, dependence on tube feeding and/or dietary supplements, and marked disruption of social functioning (5, 10). In the ARFID concept, three profiles can be recognized, such as the profile with a reduced hunger drive, a profile where children avoid food due to sensory sensitivity, and a profile where food is avoided due to fear of the aversive consequences of food/eating; the latter are often children with post-traumatic feeding or eating experiences. Thus, the selective and/or restrictive eating pattern of children with ARFID can often be traced back to the presence of one or more of these profiles

Parents of children with disordered feeding or eating patterns often seek professional assistance. However, some studies show that professionals tend to endorse the opinion that these children “will most likely outgrow their feeding problems” (11, 12). Identification of predictors of continuous disordered feeding/eating may be helpful for professionals to ground their advice for intensive treatment or not. Currently, there is limited knowledge about the natural course of disordered feeding and/or eating problems.

This retrospective study aims to provide insight into predictors associated with chronic disordered feeding or eating, which started in infancy or early childhood. Because a substantial part of the study concerns chart reviews (*t*1 variables) from 2004 to 2015, where data were collected in the scope of DSM-IV or ICD−10 (13), this study does not primarily refer to ARFID but to disordered feeding or eating. In this study we investigate the following two questions: (I) how many young children with disordered feeding or eating patterns did recover over the course of time toward an age-appropriate feeding or eating pattern, after refraining from intensive treatment, and (II) which factors predict the persistence of feeding/eating problems at a later age, when no intensive treatment was provided?

## Materials and Methods

### Participants

In total, parents of 296 children who were referred by their pediatrician, and who had entered the intake phase between 2004 and 2015, but who did not obtain treatment at SeysCentra (a tertiary treatment center for children with serious feeding or eating problems in The Netherlands), were invited for participation in this study by letter. Parents of 60 children did not participate: Two children had passed away (for other reasons than feeding problems), four had moved and could not be traced anymore, two participants with an intellectual disability were excluded because their age exceeded the limit of 18 years, and the other 52 parents refused to participate (with no specific reason). This resulted in a final sample of 236 children with disordered feeding/eating patterns [105 girls (44.5%)]. At initial consultation (*t*1), the children had an average age of 4;9 years (SD = 3;6 years; range 5 months to 17 years), and at the telephone survey (*t*2) the average age was 11;2 years (SD = 4;10 years; range 2 to 24 years). All participating children in this study were referred by a pediatrician and medically screened for possible underlying factors responsible for the eating disorder, likewise for allergies. If these were established and supervised by the dietitian, this was not an exclusion criterion for participation in treatment.

### Procedure

Each child involved and its parents were referred by a pediatrician to seek support from a tertiary treatment center for disordered feeding or eating. Upon referral, an initial consultation took place in which a physician, dietician and psychologist examined the child and questioned the parent(s) about several topics, including the variables under investigation.

All children in this study had an age inappropriate food intake (related to, e.g., tube dependency, and/or serious underweight and/or nutritional deficiencies).

Despite treatment advice, a substantial part of these children did not start treatment at the tertiary treatment center. These children's parents chose to refrain from (C)BT-day treatment for several reasons, such as their child's young age, their worries about attachment development, and/or a long travel time to the treatment center and/or expected intrusiveness of the treatment.

At a second time point (*t*2; 6 years after *t*1, on average), the parents of all 296 children who had previously chosen to refrain from treatment were approached, to take part in a study about the current status of their children's feeding/eating problems. For those who consented to participate, two independent procedures started: (I) An analysis of data that were collected at *t*1, to establish the types and levels of disordered feeding/eating patterns during the initial consultation. This was based both on the child's feeding and eating history and physical (e.g., weight, oral-tube ratio, nutritional value of food intake, vomiting) and psychosocial measures (e.g., parental distress, behavioral problems). In addition, the presence or absence of a set of variables such as gastro-intestinal problems, autism spectrum disorder, syndrome/intellectual impairment, comorbidity, was checked. (II) A standardized telephone survey with at least one parent was planned by an independent interviewer who was blinded regarding the child's specific food problems and background variables. The aim of the interview was to collect information about the child's current status in terms of disordered feeding/eating and to determine the child's food intake, with similar criteria as at *t*1. The independent interviewer was well experienced in telephone survey research. The survey took 15 min, on average.

### Design and Measurements

This cohort study was a retrospective correlational study, with measurements at two time points (*t*1 and *t*2). We aimed to establish which, if any, variables measured at *t*1 could successfully predict an age-(*in*)adequate food intake at *t*2.

*Measurements at t*1*:* All variables at *t*1 were obtained from the consultation report using a standardized rating format. First, all binary variables pertaining to a characteristic were coded “0” if absent and “1” if present (e.g., a child scored “0” on “Autism” when no (diagnosed) autistic problems were present, and “1” when (diagnosed) problems of an autistic nature were mentioned).

The following variables were recorded at *t*1:

(a) *Sex* [i.e., male (coded “0”) vs. female (coded “1”)].
(b) *Ethnicity* [i.e., Caucasian (coded “0”) vs. non-Caucasian (coded “1”)].
(c) *Prematurity/dysmaturity* (prematurity: the child was born before 37 weeks of pregnancy; dysmaturity: an intrauterine growth restriction, meaning the child had a birth weight less than 2500 g, but was born on time. The child can have either of these conditions or both. Having both is indicated as pre-dysmaturity).
(d) *Medical comorbidities* (i.e., organic failures/diseases other than gastro-intestinal problems, such as kidney disease, cardiac problems, metabolic disease).
(e) *Suffering from any gastrointestinal disease* (e.g., gastro-esophageal reflux disease (GERD), or celiac disease, or delayed gastric emptying, or food allergy).
(f) *Having a delineated genetic syndrome* (i.e., a syndrome with phenotypical feeding disturbances, e.g., Down syndrome, Angelman syndrome, Silver Russel syndrome, Noonan syndrome, and/or being *intellectually impaired* with an Intelligence Coefficient (IQ) < 70).
(g) *Diagnosed with Autism Spectrum Disorder (ASD)*.
(h) *Having a history of age-appropriate food-intake* (i.e., whether the child had a normal feeding or eating pattern before developing its feeding difficulties).
(i) *Age*.
(j) *Latency* time (in years) between *t*1 and *t*2.

Next, we reviewed variables that were specifically related to the food intake problems, such as parents' estimation of the child's feeding or eating performance at *t*1. This score could range from “1” (i.e., ‘child refuses any orally presented food', ‘requiring tube feeding') to “12” (“child eats fully orally and accepts food items with a solid texture”), on a 12-item rating scale that was derived from the Seyshuizen Food Refusal Questionnaire (SFRQ) (14). In Table 1 the scores are listed. The SFRQ, which is validated for the Dutch population, turned out to have good psychometric characteristics. The SFRQ includes 3 subscales: (1) “intrusiveness,” (2) “texture,” and (3) “variation”. Inter-rater reliability was significant with percentages of agreement ranging from 83–100 (subscale 1); 77–100 (subscale 2) and 97–100 (subscale 3), with accompanying Kappas ranging from.66–1.00 (subscale 1);.54–1.00 (subscale 2) and.94–1.00 (subscale 3), respectively. Intra-rater reliability was comparable, with percentages of agreement ranging from 86–100 (subscale 1); 83–100 (subscale 2) and 89–100 (subscale 3), with Kappas of.72–1.00;.66–1.00 and.78–1.00, respectively. Spearman correlation coefficients between inter- and intra-rater reliability ranged from *r* = 0.88 to *r* = 0.97) with all accompanying *p*-values < 0.001. “Predictive validity” was determind by means of assigning significant lower subscale scores from the rater's judgement (group 1), as compared to the respondent's judgement (group 2). Differences were estimated using a non-parametric Man-Whitney test who showed *U*–values of 240.5, 181.0, and 340.5, all with *p*-values of < 0.001, which can be considered as very satisfactory. The same applies to the concurrent validity, with *p*-values of < 0.001, after calculating Spearman correlation between the rankings of the SFRQ-rates for the various subscales and the rankings provided by an independent observer of the video recordings during the mealtimes, with values ranging from *r* = 0.95 to *r* = 0.99 (14).

**Table 1:** Seyshuizen food refusal questionnaire (SFRQ) items rated at consultation (*t*1) and telephone survey (*t*2).

1. Child refuses any orally presented food, requires tube feeding
2. Child's food intake consists entirely of liquid medical nutrition, orally taken
3. Child's food intake consists partly of orally presented liquid foods, and partly of tube feeding
4. Child's food intake consists partly of orally presented pureed foods, and partly of tube feeding
5. Child's food intake consists partly of orally presented mashed foods, and partly of tube feeding
6. Child's food intake consists partly of orally presented mixed mashed foods, and partly of tube feeding
7. Child's food intake consists partly of food items with a solid texture, and partly of tube feeding
8. Child's food intake is fully oral, though fully liquid
9. Child's food intake is fully oral, though fully pureed
10. Child's food intake is fully oral, though fully mashed
11. Child's food intake is fully oral, including mixed mashed foods
12. Child eats fully orally and accepts food items with a solid texture

Lastly, ratings for the following binary variables “Restrictive Caloric Food Intake (RCFI),” “Selective Food Intake” (SFI), “Lack of Varied Nutritional Intake” (LOVNI), and “Selective Texture Choices” (STC) were coded “0” when absent and “1” when present. The parents' reports on these variables were first rated and later converted into scores by two qualified researchers, independently. The category “Restrictive Caloric Food Intake” (RCFI) was scored positive if the child was (partly) tube–dependent and/or was significantly underweight and/or used medical nutrients to provide his daily quantity of an age-appropriate caloric intake, in accordance with the Dutch Youth Health Care Guidelines in Eating and Feeding Behavior (15). A “Selective Food Intake” (SFI) indicates (I) a child who repeatedly refused (nearly all) food items from one or more categories from the five basic food groups (of the Dutch “Schijf van 5”, which is an equivalent of “MyPlate”) without using any compensation (such as in the case of vegetarian eating) and/or (II) who repeatedly refused food items with a specific texture or bite. The behavior was labeled as “Lack of Varied Nutritional Intake” (LOVNI) when the acceptance per food group was too low, e.g., eating just one or a few bites of one or two types of vegetables or fruits and after calculation the minimum required nutritional value was not achieved. Avoiding food items for medical reasons, such as allergies, was excluded. When children repeatedly refused certain texture(s), e.g., only eating grinded or liquid foods, this was labeled as “Selective Texture Choices” (STC). Based on the reviewed data it was then determined whether the child could be classified and confirmed as having an age-appropriate food intake (code “0”), or an age-inappropriate food intake (code “1”) at *t*1.

*Measurements at t*2: The variables at *t*2 were assessed in a telephone survey which was formalized in a standardized flow-chart provided for the data collection, to determine whether the child had currently an age-appropriate food intake. First, the parents were asked to rate their child with the most representative score, at that moment, on the 12 item-SFRQ and secondly, they were interrogated about their child's selective and/or restrictive food intake, while the interviewer rated this as present (“1”) or not (“0”), using the same criteria as at *t*1. Afterwards, the collected data were entered in a data file and, in a similar vein as the *t*1 data, it was determined whether the child could be classified as having an age-appropriate food intake (code “0”), or age-inappropriate (code “1”) at *t*2.

### Statistical Analysis

All statistical analyses were performed using SPSS 24. To examine the sample characteristics, we first conducted univariate analyses by calculating frequencies, means and standard deviations of the background variables, presence or absence of pre/dysmaturity, gastro-intestinal disease, history of age-appropriate feeding, syndrome/developmental impairment, autism spectrum disorder, comorbidity, and the child's age and sex, at *t*1, followed by frequencies and percentages of the dependent variables “RCFI,” “SFI,” “LOVNI,” “STC”, and “AAFI,” at *t*1 and *t*2. Then, we calculated correlations between these variables to determine whether there were any significant negative or positive associations between these variables within the sample. Next, a binary logistic regression was conducted to assess whether one or more of the background variables proved predictive of an age (*in*-)appropriate food intake at *t*2. In order to restrict the probability of missing potentially interesting predictors, and due to the large number of predictors we used an alpha of 0.10 for establishing significance. This adjustment of the conventional significance level, which is common in exploratory backwards regression analyses, was chosen to decrease the probability of type II errors.

## Results

### Description of the Sample

At *t*1, 18.6% of the sample (44 children) could be described as born prematurely and/or dysmaturely. Some had spent time in the incubator or had been tube dependent for a while. Forty-seven percent (111 children) could be classified as suffering from comorbidity with organic diseases other than gastro-intestinal disease, such as kidney disease, cardiac problems and metabolic disease. A specific syndrome or intellectual disability (IQ < 70) was present in 57 (24.2%) children. About 56 (23.7%) of the children were familiar with ASD and had received an official diagnosis by a previous care provider. Another substantial part of the sample [56.8% (134 children)] had gastro-intestinal problems such as gastro-esophageal reflux disease (GERD), celiac disease, delayed gastric emptying, or food allergy at *t*1. Only 18.5% of the sample had a history of an age-appropriate food intake and started their feeding difficulties only after a period with a normal health and feeding pattern. A restrictive caloric food intake (“RCFI”) was observed in 67.4% of the sample, while a general selective food intake (“SFI”) was present in 96.6%. Within the “SFI” group, 82.2% showed a lack of varied nutritional intake (“LOVNI”) intake and 75.4% showed (also) selective texture choices (“STC”). At *t*1, logically, none of the 236 children showed an age-appropriate food intake (“AAFI”). At *t*2, however, 37% of these children had acquired an age-appropriate pattern without receiving any treatment. Table 2 displays frequencies and percentages of variables used to classify a child as age-appropriate, or not. The table shows the differences in frequencies between *t*1 and *t*2 in food selectivity [(a) “LOVNI” and (b) “STC” and (c) “SFI” (general score)], “RCFI,” and an age adequate food intake (“AAFI”). It appears from the SFRQ measures that feeding skills (related to the need for tube feeding and texture differentiation), in general, improved over time within the sample. The mean score at *t*1 was “7,” and at *t*2 was “10”. This suggests a decrease in (partly) tube feeding dependency, indicating an improvement in oral acceptance and somewhat in texture differentiation. At *t*1, 58 children (24.4%) obtained a score of 1 on the SFRQ, indicating “Child refuses any orally presented food, requires tube feeding,” while at *t*2 this score was observed in only 16 (6.8%) of the children. An opposite shift was observed regarding item 12 (“Child eats fully orally and accepts food items with a solid texture”). At *t*1, score “12” was applicable in only 62 children (26.1%), but at *t*2 in 152 children (64.4%).

**Table 2:** Comparison of frequencies and percentages of dependent variables between *t*1 and *t*2 (*N* = 236).

	**Frequency *t*1**	**Percentage**	**Frequency *t*2**	**Percentage**
**Restrictive caloric food intake (RCFI)**				
Absence	77	32.6	161	68.2
Presence	159	67.4	75	31.8
**Selective food intake (SFI)**				
Absence	8	3.4	104	44.1
Presence	228	96.6	132	55.9
**Lack of varied nutritional intake (LOVNI)**				
Absence	42	17.8	120	50.8
Presence	194	82.2	116	49.2
**Selective texture choices (STC)**				
Absence	58	24.6	160	67.8
Presence	178	75.4	76	32.2
**Age-appropriate food intake (AAFI)**				
Absence	236	100	148	62.7
Presence	0.00	0.00	88	37.3

### Inter-rater Reliability of the Assessment of the Survey Results

A random selection of 25 surveys (10.6 % of all surveys), was independently scored by 2 raters. These rated both the posttest SFRQ scale scores (1–12) and the binary variables (0–1): “SFI,” “LOVNI,” “STC,” “RCFI” and “AAFI” from the information obtained from the telephone survey (*t*2). To compile a sample representative group, 2–3 surveys from each year of consultation (between 2004 and 2015), were randomly chosen by an employee who was not involved in the research. Inter-rater reliability was determined by calculating Cohen's Kappa. A Kappa of 1.0 was obtained, indicating perfect agreement. Of the 150 ratings observed within the 25 surveys, the two independent raters only disagreed about 1 rating. This rating was discussed.

### Correlations

Bivariate analysis shows several significant correlations at the 0.01 and 0.05 level (two-tailed), although all can be typified as small to medium. We will summarize the relevant correlations. A negative correlation was found between “age of the child at *t*1” and “AAFI” at *t*2 (−0.217, *p* = 0.001), indicating a reduced chance of spontaneous recovery if the child was older at the time of initial consultation. Another significant (negative) correlation was found between “LOVNI” and “AAFI” at *t*2 (−0.214, *p* = 0.001), meaning that children with a less varied food intake at *t*1 more often failed to develop an age-appropriate food intake at a later age. A third significant (negative) correlation regarding an “AAFI” at *t*2 was found with “ASD diagnosis” (−0.204, *p* = 0.002), suggesting that participants with ASD less often developed an age-appropriate feeding pattern at *t*2. A fourth significant (positive) correlation was found between “latency time between consultation (*t*1) and survey (*t*2)” and an “‘AAFI” at *t*2 (0.129, *p* = 0.047), which suggest that the chance to develop an age-appropriate food intake increases over time. A final significant correlation was found related to sex (0.174, *p* = 0.008), indicating that girls more often showed an age-appropriate food intake at *t*2 than boys, thus boys having a poorer prognosis. Striking, and perhaps not expected, was that the SFRQ pretest scale score at *t*1 hardly and insignificantly correlated with “AAFI” at *t*2 (−0.075; *p* = 0.249). The score distribution of this adapted 12-item SFRQ appeared somewhat bimodal with one of the tops close to the maximum rate of 12. Table 3 provides an overview of the correlations between all *t*1 measures and an AAFI at *t*2.

**Table 3:** Correlations between *t*1 measures and age-appropriate food intake (AAFI) at *t*2.

	**AAFI (** ***t*** **2)**	
***N =* 236**	Correlation^***^	**Sig. (2-tailed)**
Age (*t*1)	−0.217^**^	0.001
Latency (*t*1–*t*2)	0.129^*^	0.047
Sex	0.174^**^	0.008
ASD	−0.204^**^	0.002
Pre/Dysmature	−0.054	0.408
GIP	0.089	0.173
Comorbidity	0.039	0.548
Syndrome/ID	−0.074	0.259
History of AAFI	0.114	0.080
RCFI (*t*1)	0.051	0.438
SFI (*t*1)	−0.071	0.277
STC (*t*1)	−0.008	0.908
LOVNI (*t*1)	−0.214^**^	0.001
SFRQ (*t*1)	−0.75	0.249

* *Correlation is significant at the 0.05 level (2-tailed)*.

** *Correlation is significant at the 0.01 level (2-tailed)*.

*** *Correlations between two continuous variables are linear (Pearson) correlations. Correlations between continuous variables and binary variables are point biserial correlations. Correlations between two binary variables are Phi correlations*.

*GIP, Gastro-Intestinal Problems; ID, Intellectual Disability; RCFI, Restrictive Caloric Food Intake; SFI, Selective Food Intake; STC, Selective Texture Choices; LOVNI, Lack Of Varied Nutritional Intake; SFRQ, Seyshuizen Food Refusal Questionnaire; t1, time point 1; t2, time point 2*.

### Predicting Age-Appropriate Food Intake at *t*2

The variable Age Appropriate Food Intake (AAFI) was taken as the outcome measure, and all other variables as the predictors. Age-appropriate food intake implies no significant disturbances in the necessary caloric food intake, a varied nutritional intake and a presentation of all textures in the daily meals. In Table 4, all *t*1 variables are summarized with relevant Odds Ratios (OR). A number of variables turned out to be significant predictors. First, age at *t*1, with a seemingly very small OR of 0.991. For continuous predictors, the magnitude of the regression coefficient is scale dependent and cannot be interpreted in terms of effect size. The value of −0.01 implies a substantial change in the odds of an age adequate food intake pattern for children who differ 4 years in age, for instance. So, this implies that children with a younger age at *t*1 have a decreased chance to remain in an age-inappropriate food intake. Another predictor was found in “latency between *t*1–*t*2” with an OR of 1.008 which means that a longer duration between *t*1 and *t*2 predicts an age adequate food intake. Also “sex,” with an OR of 1.789, appeared significant. This means that boys are at greater risk to remain in a disordered feeding or eating pattern. “Autism Spectrum Disorder diagnosis,” with an OR of 0.458, also turned out to be predictive for the absence of an AAFI at *t*2, as well as the selective eating variables “STC” with an OR of 0.392, and “LOVNI” with an OR of 0.303. Thus, selective eating at younger ages appears a serious risk factor for not developing an AAFI. In Table 4, all the other covariates with ORs and corresponding Logodds are displayed. A final validation in possible confounding variables by providing a collinearity check showed no problematic overlap in predictors.

**Table 4:** Logistic regression model of all *t*1 variables in the equation.

	**B (LogOdds)**	**Wald (df = 1)**	**Sig.(*p*)**	**Odds ratio**	**95% C.I. (OR)**	
					**Lower**	**Upper**
Age (*t*1)	−0.010	4.946	0.026	0.991	0.982	0.999
Latency *t*1–*t*2	0.008	4.582	0.032	1.008	1.001	1.016
Sex	0.582	3.471	0.062	1.789	0.970	3.299
Pre-dysmaturity	−0.356	0.848	0.357	0.700	0.328	1.495
GIP	0.104	0.090	0.764	1.110	0.563	2.186
Comorbidity	0.006	0.000	0.984	1.006	0.548	1.849
Syndrome/ ID	−0.198	0.289	0.591	0.820	0.399	1.688
ASD	−0.782	2.902	0.088	0.458	0.186	1,125
History of AAFI (*t*1)	0.181	0.152	0.697	1.198	0.483	2.975
SFRQ (*t*1)	−0.053	0.839	0.360	0.948	0.847	1.062
RCFI (*t*1)	−0.534	1.426	0.232	0.586	0.244	1.409
STC (*t*1)	0.935	3.826	0.050	0.392	0.154	1.002
LOVNI (*t*1)	−1.196	8.702	0.003	0.303	0.137	0.669
Constant	1,576	1.775	0.183	4.834		

*GIP, Gastro-intestinal problems; ID, Intellectual Disability; ASD, Autism Spectrum Disorder; AAFI, Age-Appropriate Food Intake; SFRQ, Seyshuizen Food Refusal Questionnaire; RCFI, Restrictive Caloric Food Intake; SFI, Selective Food Intake; STC, Selective Texture Choices; LOVNI, Lack of Varied Nutritional Intake; OR, Odds Ratio; df, degree of freedom; C.I., Confidence Interval*.

As displayed in Table 4, some of the effects in the regression model are not significant, and, thus, unjustly corrected for overlap. Therefore, we added an extra (Table 5) to show the significant effects corrected only for the relevant effect between the predictors.

**Table 5:** Logistic regression model after sequential deletion of non-significant predictors (Alpha = 0.10).

	**B (LogOdds)**	**Wald (df = 1)**	**Sig.(*p*)**	**Odds ratio**	**95% C.I. (OR)**	
					**Lower**	**Upper**
Age (*t*1)	−0.010	6.639	0.010	0.990	0.982	0.998
Latency *t*1–*t*2	0.009	5.102	0.024	1.009	1.001	1.016
Sex	0.529	3.002	0.083	1.698	0.933	3.090
ASD	−0.749	3.107	0.078	0.473	0.206	1.087
STC (*t*1)	−0.625	2.804	0.094	0.536	0.258	1.112
LOVNI (*t*1)	−1.128	8.727	0.003	0.324	0.153	0.684
Constant	0.709	1.494	0.222	2.031		

*ASD, Autism Spectrum Disorder; STC, Selective Texture Choices; LOVNI, Lack Of Varied Nutritional Intake; OR, Odds Ratio; C.I., Confidence Interval*.

## Discussion

This study aimed to identify predictors of age-inappropriate food intake at a later age in young children who were diagnosed with disordered feeding or eating but refrained from intensive treatment. This was done by a retrospective research design, where several factors were investigated at two different time points, with - on average - 6 years in between. Main findings were as follows: only slightly more than one third (37%) of the young children with disordered feeding or eating patterns had an age-appropriate food intake at a later age - without having engaged in specialized treatment. Hence, almost two third (63%) of the children *still* showed disordered feeding or eating patterns that could most likely be regarded as ARFID. In general, we observed a decrease in disordered feeding behavior at follow-up (*t*2) related to the first consultation (*t*1) within the variable's restrictive caloric food intake (RCFI: *t*1 = 67, 4%/*t*2 = 31, 8%), selective food intake (SFI: *t*1 = 95, 6%/*t*2 = 55, 9%) and age-inappropriate food Intake (*t*1 = 100%/*t*2 = 63%). Regarding the SFI measures we observed a decrease in the “selective texture choices” variable (STC: *t*1 = 75, 4%/*t*2 = 32, 2%) and a decrease in the “Lack of varied nutritional intake” variable (LOVNI: *t*1 = 82, 2%/*t*2 = 49, 2%) which suggests that eating a small range of varied meals at a young age is even more difficult to change than texture problems. These findings are in line with the results of Kotler and colleagues (16), who performed a 17-year longitudinal study of eating problems from childhood through adulthood. The authors suggest that eating disorder symptoms are stable over time which implies a relationship between early childhood feeding problems and the development of eating disorders later in life. The study revealed that childhood feeding problems with restrictive and selective eating patterns increased the risk for developing anorexia nervosa (AN) later in life (16). Although AN is based on a different etiologic concept (distorted body image), it is interesting to compare them, as restrictive eating behavior can be a common symptom; in fact, restriction in ARFID can also be of a selective nature.

Based on our outcomes we could inform parents, GPs and pediatricians about the likeliness of spontaneous recovery of serious feeding/eating difficulties in only one third of the children. In addition, the outcomes on the 12-item SFRQ showed an improvement from 7 (“Child's food intake consists partly of food items with a solid texture, and partly of tube feeding”) to 10 (“Child's food intake is fully oral, though fully mashed”). So, most of the children probably recover over time from tube feeding, but only partly recover in selective feeding (in this scale only measured as texture selectivity). In other words, being an extremely selective eater at a young age might indicate a worse prognosis than being an extremely restrictive eater (or even a tube-dependent child), when refraining from intensive treatment.

Furthermore, we checked several potential predictors that might hinder spontaneous recovery at a later age by using logistic regression. The observed significant association between age of the child at initial consultation and having an age-appropriate intake at *t*2, indicates a reduced chance of spontaneous recovery if the child is older at initial consultation, which is hard to explain. As hypothesized, a neophobia may explain the reduced chance of spontaneous recovery when the child is older. In other words, when the child is younger at *t*1, its selective eating is more likely to be due to an age-related temporary neophobia which might, thus, be overcome at *t*2. Still, it could also implicate that sooner referral of the child will increase its chances of recovery, even if no intensive treatment is advised. A second significant association was found between the extent to which a child eats varied (denoted as “LOVNI”) at a young age and having an age-inappropriate food-intake at later age. That is, eating less varied at a young age is associated with a smaller chance of age-appropriate eating at a later age. This suggests that sticking to a child's food preferences (e.g., meals lacking vegetables and/or fruits or largely consisting of crunchy/ fried foods), and avoiding confrontations at early age with varied meals, provides a poorer prognosis.

The association between sex and problematic eating at a later age, suggesting that boys are less likely to develop an age-appropriate food intake than girls, is somewhat remarkable. This is in contrast with other eating disorders, like anorexia/bulimia nervosa, from which women suffer considerably more. Another significant predictor is ASD diagnosis at *t*1. A literature review from Ledford and Gast (17) indicates that over half of the children with ASD have behavioral or nutritional concerns related to feeding and a meta-analysis (18) found that children with ASD were about five times more likely to experience a feeding problem compared to children without ASD. This implies that children with ASD are at risk for developing disordered feeding patterns (19). It's hypothesized that sensory sensitivity may influence the selective and restrictive eating patterns in these children. Food selectivity indeed appears to be a significant issue for many of these children (20, 21). Nadon and colleagues (22) examined the relationship between problems of sensory processing and several eating problems in children with ASD. Of the 95 investigated children with ASD (aged 3 to 10 years), 65% percent showed a definite difference and 21% a probable difference in sensory processing on the total score of the “Short Sensory Profile” (23, 24). The results were significantly related to an increase in the number of eating problems measured by the “Eating profile” (25). A second hypothesis for the pervasive course could be that high sensitivity for developing psychotrauma and the lack of a theory of mind, limits abstract reasoning and cognitive change. Children with ASD are, indeed, often limited in developing adequate coping skills to respond to significant stressors. So, tasting new unknown flavors, or deviations in textures, or having other expectations of taste and/or textures of certain food items might be stressful for these children.

A substantial part of the sample had a physical comorbidity, like Gastro Intestinal Problems (GIP), which partly involved medication, with the aim of minimizing physical complaints. Children were only eligible for referral to the tertiary center if the physical complaints were being managed/ under control and were no longer a contra-indication to learning to eat or learning to control their eating anxiety. In the end, medication-use was not included as a variable to investigate. Afterwards we concluded that having GIP was *not* predictive for remaining in an age-Inadequate food intake at later age.

In order to interpret these results properly, we should also consider the limitations of this study. Firstly, the use of binary variables instead of - for instance - Likert type scales which might show more variability. The choice for these types of variables was directly related to the available chart review data at *t*1. Furthermore, there was a lack of proper, more differentiating, valid measurements for young children during that time period (start 2002), which were comparable with contemporary diagnostic ARFID-tools. Instead, we preferred a larger sample size, while collecting binary data from chart reviews by recording the presence or absence of relevant variables by a standardized format. A second limitation is the heterogeneity of the sample which makes generalization more nuanced. We applied a logistic regression model including collinearity analysis on possible confounding variables concerning typical sample characteristics, e.g., “ASD,” “Syndrome/ID,” “Sex,” “Age,” “Ethnicity,” organic issues etc. There was no problematic overlap found. Therefore, we endorse the opinion that these heterogeneities could also be a strength. The sample concerns a cross-section of a treatment population referred to a tertiare clinic for severe feeding & eating problems. Selection and exclusion to the sample on typical characteristics might infringe the representativeness. Analyzing disordered feeding patterns in (young) children by chart reviews, before 2013, might lead to ambiguity in defining the appropriate diagnostic terms, compared to those after 2013. And the same goes for DSM-related diagnostic tools and questionnaires for determining disordered feeding. However, in both classifications, disordered restrictive and/or selective eating patterns are the starting point, and the associated criteria to be met show great similarity. We would also like to point out that Seyscentra was already a tertiary center before 2013, to which only children with serious pediatric feeding/eating problems could be referred by a pediatrician.

On the basis of our dataset which was not evenly distributed in age when it comes to pre- or peri-adolescents, it was difficult to determine whether peri-adolescents recover more or less often on their own. And that also applies to making statements about this on the basis of gender to this age groups. This was not taken into account in advance when composing the sample, because all children who registered and had not received treatment were approached.

As concluded earlier, there is limited knowledge about the natural course of disordered feeding and/or eating problems The most obvious way to investigate this would be by a longitudinal study design in which one group of patients receives intensive treatment while the other does not. However, ethical objections logically hinder such an approach; therefore, we have attemped an interesting alternative, by applying a retrospective study in a group of children who presented with disordered feeding, but who did not receive treatment.

We chose for the SFRQ as a supportive measure for disordered feeding, by determining tube feeding independency, texture choices and varied food intake. SeysCentra developed and studied the SFRQ which turned out to be reliable and valid in the Dutch population. It is, therefore, still in use as a diagnostic tool in our clinic (26). Although the SFRQ is based on the DSM-IV/ICD-10 classification and only available in Dutch, the questionnaire is still used in clinical centers because it quite adequately assesses the disordered feeding behaviors, we are interested in. Lastly, we realize that a substantial part of participating children who initially refrained from (cognitive-)behavioral treatment have possibly had other forms of assistance between *t*1 and *t*2. Unfortunately, this has not been systematically checked and therefore appropriate insight into this aspect is lacking. Despite this, two third of the sample did not reach an age-appropriate food intake.

The confirmation from the logistic regression that ASD can be classified as a predictor for remaining in an age-inappropriate food intake, as well as previous findings in the literature makes ASD probably one of the most relevant determinants and should, to our opinion, be the subject of early intervention. Besides the determinants which might predict a lifelong problematic feeding pattern, evidence was found for the assumption that food selectivity, when the child refrains from a varied food intake at younger age, barely leads to spontaneously recovery. This makes selective food acceptance a persistent and probably one of the most underestimated types of disordered feeding.

This study attempts to answer whether it is justified to advice the parents of young children with disordered feeding, to “wait and see.” Despite the general improvements over time in around two third of the children, they still demonstrated an age-*in*appropriate food intake. Therefore, we would advise health care professionals to take special notice of certain risk groups. These include children with ASD and children with an extremely selective food intake, in particular boys. We also want to emphasize that the psychosocial consequences associated with disordered feeding and eating should not be underestimated. Treatment could probably reduce these considerably. Despite the fact that larger RCT's on treatment of disordered feeding are ongoing, several case series reported on (C)BT as a proper treatment for disordered feeding. (C)BT for young children and adolescents with disordered feeding or eating is quite intensive but effective (24, 27–32).

Despite that, further research on treatment in young children, in particular those at risk for long-term feeding problems, should be conducted to see whether providing timely and appropriate treatment indeed leads to improvement.

## Data Availability Statement

The raw data supporting the conclusions of this article will be made available by the authors, without undue reservation.

## Ethics Statement

The study was approved by the Ethics Review Committee of the Faculty of Psychology and Neuroscience (ERCPN), Maastricht University (ERCPN-189_07_03_2018). Written informed consent to participate in this study was provided by the participants' legal guardian/next of kin.

## Author Contributions

ED carried out the study and participated in the design, data collection, data analysis, and wrote the first draft of the manuscript. AJ supervised and assisted in all phases of the study, including providing feedback on drafts of the manuscript. SM and NB participated in the design, data collection, data analysis and subsequent versions of the manuscript. PD and DS assisted in carrying out the study and data analysis. All authors contributed to the article and approved the submitted version.

## Conflict of Interest

The authors declare that the research was conducted in the absence of any commercial or financial relationships that could be construed as a potential conflict of interest.

## Publisher's Note

All claims expressed in this article are solely those of the authors and do not necessarily represent those of their affiliated organizations, or those of the publisher, the editors and the reviewers. Any product that may be evaluated in this article, or claim that may be made by its manufacturer, is not guaranteed or endorsed by the publisher.

## Abbreviations

APA: American Psychiatric Association
ARFID: Avoidant/restrictive food intake disorder
AAFI: Age appropriate food intake
ASD: Autism spectrum disorder
(C)BT: (Cognitive-)behavioral therapy
DSM-IV-TR, Diagnostic and statistical manual of mental disorders, fourth edition: Text Revision
DSM-5, Diagnostic and statistical manual of mental disorders: fifth edition
GIP: Gastro-intestinal problems
ICD-10, International Classification of Diseases and Related Health Problems: tenth edition
ID: Intellectual Disability
LOVNI: Lack of varied nutritional Intake
RCFI: Restrictive caloric food intake
SD: Standard Deviation
SFI: Selective food intake
SFRQ: Seyshuizen food refusal questionnaire
SP: Sensory profile
SPP: Sensory processing problems
STC: Selective texture choices
Syndr/ID: Syndrome and/or intellectual disability
*t*1: time point 1
*t*2: time point 2.
